# Design Optimization and Comparison of Cylindrical Electromagnetic Vibration Energy Harvesters

**DOI:** 10.3390/s21237985

**Published:** 2021-11-30

**Authors:** Tra Nguyen Phan, Jesus Javier Aranda, Bengt Oelmann, Sebastian Bader

**Affiliations:** Department of Electronics Design, Mid Sweden University, 85170 Sundsvall, Sweden; tra.phan@miun.se (T.N.P.); javier.aranda@miun.se (J.J.A.); bengt.oelmann@miun.se (B.O.)

**Keywords:** vibration energy harvesting, electromagnetic energy harvesting, electromagnetic coupling coefficient, design optimization, expensive black-box optimization

## Abstract

Investigating the coil–magnet structure plays a significant role in the design process of the electromagnetic energy harvester due to the effect on the harvester’s performance. In this paper, the performance of four different electromagnetic vibration energy harvesters with cylindrical shapes constrained in the same volume were under investigation. The utilized structures are (i) two opposite polarized magnets spaced by a mild steel; (ii) a Halbach array with three magnets and one coil; (iii) a Halbach array with five magnets and one coil; and (iv) a Halbach array with five magnets and three coils. We utilized a completely automatic optimization procedure with the help of an optimization algorithm implemented in Python, supported by simulations in ANSYS Maxwell and MATLAB Simulink to obtain the maximum output power for each configuration. The simulation results show that the Halbach array with three magnets and one coil is the best for configurations with the Halbach array. Additionally, among all configurations, the harvester with two opposing magnets provides the highest output power and volume power density, while the Halbach array with three magnets and one coil provides the highest mass power density. The paper also demonstrates limitations of using the electromagnetic coupling coefficient as a metric for harvester optimization, if the ultimate goal is maximization of output power.

## 1. Introduction

In recent years, the concept of the internet of things (IoT) has developed rapidly in various fields [1,2,3]. The main concept is to enable the interconnection of physical infrastructures and devices in order to obtain valuable information typically generated by sensors. In such a system, the monitoring and communication can be achieved via distributed sensor networks. These networks not only monitor and record real-time information regarding the connected devices, but also forward the collected data to a central unit for storage and further processing. Due to easier deployment and a greater level of flexibility, wireless sensor networks (WSNs) are a key technology for IoT implementation [4]. However, one of the main challenges when dealing with wireless sensor nodes is their power supply. Wired power supplies inhibit the advantages gained by wireless connectivity, and batteries generate additional maintenance and disposal needs. To overcome these problems, energy harvesting has been identified as a power supply alternative worth investigating [5,6,7].

Energy harvesting refers to the process of converting ambient energies into electrical energy to power electronic devices [8]. Energy harvesting can be classified based on the ambient energy source, e.g., mechanical energy, thermal energy, radiant energy, and chemical energy. Since mechanical vibrations are abundant in the environment of many applications, it has attracted many investigations and research studies [9]. Vibration energy harvesting can further be categorized into three main types based on the conversion mechanism used. These conversion mechanisms are electromagnetic [10], piezoelectric [11] and electrostatic transduction [12]. Compared to electrostatic energy harvesters, electromagnetic transducers are more suitable for large-scale applications [13]. They also do not require an external voltage bias to get started. Moreover, electromagnetic transducers are typically more robust and economical than piezoelectric transducers.

Electromagnetic energy harvesting [14,15] utilizes relative motion between a coil and a magnetic field, which results in the time variation of the magnetic flux. According to Faraday’s law, the changing flux will induce a voltage across the coil. We therefore believe that the investigation of the magnet and coil structure plays an important role in the design process of an electromagnetic energy harvester.

The design of the magnet and coil structure has been implemented in many different ways. In terms of material, neodymium iron boron (NdFeb) has been shown as a strong magnetic material and, thus, has been used for permanent magnets many previous works [16,17]. In terms of structural design, various architectures, including single magnet [18,19,20] and magnet arrays [21,22,23] have been proposed. Among energy harvesters with magnet arrays, Halbach arrays have been an attractive topic of investigation due to their ability to concentrate the magnetic field [24,25].

The effect of the coil–magnet configuration on the performance of electromagnetic energy harvesters has also been investigated in previous studies. Spreemann et al. [26] classified different coil and magnet architectures into groups of “magnet in-line coil” and “magnet across coil”, and optimized individual structures for the output power and voltage. Zhu et al. [27] studied the performance improvement of the Halbach array with triangular cross-sectioned magnets and the double Halbach array compared to the standard Halbach array. Li et al. [28] investigated the effect of magnet shape and their arrangement on the harvester performance through four types of configurations. These are cubic-Halbach, cubic-alternative, triangle-Halbach, and triangle-alternative. Moreover, Kim et al. [29] optimized harvesters with cylindrical shape and compared the output voltage and output power according to various aspect ratios. These studies have shown that the coil–magnet topology has a great impact on the electromagnetic energy harvester’s performance.

Although the use of cylindrical shaped structures in electromagnetic harvester prototype has been popular [18,30,31], and there exist many previous studies on Halbach arrays, only few research studies have concentrated on the effect of the magnetic structure with cylindrical Halbach arrays [32,33]. Shahosseini et al. [32] presented two cylindrical magnetic structures with Halbach arrays and compared their performance with single magnet configurations and configurations of two opposite polarized magnets. However, the volume of these configurations was not constant in the comparison, but increased with the increase of the number of components (e.g. number of magnets and coils). Moreover, when conducting the power comparison between all structures, only the double-concentric Halbach array structure was geometrically optimized in terms of power density. Ordoñez et al. [33] analyzed various cylindrical configurations with Halbach arrays and compared them in terms of the magnetic flux linkage and transduction factor. However, the study lacks a comparison in terms of output power. The individual structures have also not been optimized prior to the comparison. Consequently, previous studies comparing cylindrical electromagnetic vibration energy harvesters have limitations, with respect to fair comparisons, which also limits the conclusions to be drawn from the obtained results.

In this paper, the research objective is to investigate and compare the effect of different cylindrical magnetic structures on the harvester performance in a more systematic and fair manner. This is achieved by the introduction of a fully automatic optimization procedure, which simplifies the optimization work and provides a significant output power improvement. Specifically, the performance of different harvester configurations with cylindrical Halbach arrays are compared to each other and compared to a configuration of two opposite polarized magnets separated by a spacer. In order to conduct a fair comparison, all the structures are optimized within a given volumetric constraint. The design optimization approach is based on an expensive black-box optimization routine, with the aim to maximize the output power of the electromagnetic energy harvester. The optimization takes into consideration the magnetization directions, as well as dimensions and locations of electromagnetic components.

The remainder of the paper is organized as follows. Section 2 presents the theoretical background of the electromagnetic energy harvester in general and the coil–magnet structure in detail. Section 3 explains the optimization procedure used in this study. The simulation results and discussions are shown in Section 4. Finally, Section 5 concludes the paper.

## 2. Background

### 2.1. Electromagnetic Energy Harvester Model

**Figure 1:**
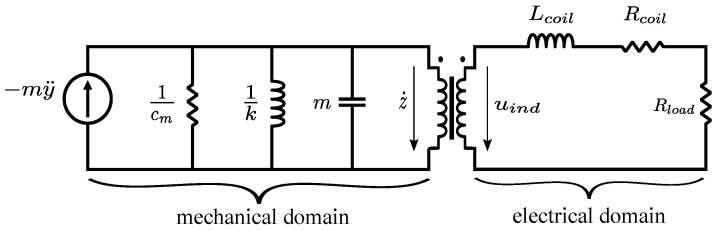
Equivalent circuit model of an electromagnetic energy harvester, including representation of the mechanical and electrical domain.

The pickup unit of an electromagnetic energy harvester consists of two main parts—permanent magnets and induction coils. When the harvester is subjected to an ambient vibration, the relative displacement between the magnets and the coil changes. According to Faraday’s law of induction, the system will induce a voltage, which can be used to power an electrical load. The equivalent circuit model of the electromagnetic vibration energy harvester connected to a resistive load $R_{load}$ is shown in Figure 1. The circuit models the mechanical as well as the electrical domain of the transducer. The governing differential equations of this electromagnetic energy harvester are described as follows

(1) $$m\ddot{z}+c_{m}\dot{z}+kz+Ki=-m\ddot{y},$$

(2) $$L_{coil}\dot{i}+\left(R_{coil}+R_{load}\right)i=K\dot{z},$$

where *m* is the inertial mass, *z* is the relative displacement between the mass and the spring, $c_{m}$ is the mechanical damping constant, *k* is the spring stiffness, and *y* is the absolute displacement of the device. $L_{c}$, $R_{coil}$, *K* are the coil inductance, coil resistance, and electromagnetic coupling coefficient respectively. *i* is the current flowing through the coil. The natural resonant frequency of the system $\omega_{n}$ is defined by the mass and the spring stiffness as

(3) $$\omega_{n}=\sqrt{k/m},$$

while the mechanical damping constant is determined by

(4) $$c_{m}=2m\zeta_{m}\omega_{n},$$

where $\zeta_{m}$ is the mechanical damping ratio. In the electrical part, the induction voltage $u_{ind}$ plays a role as the voltage source. At the lower frequencies, the effect of the coil induction $L_{coil}$ can be neglected in Equation (2). In that case, the current *i* can be solved as

(5) $$i=\frac{K\dot{z}}{R_{coil}+R_{load}}$$

Inserting Equation (5) into Equation (1) shows that an additional electrical damping constant $c_{e}$, which reflects the interaction of the induction current on the mechanical part of the harvester, can be expressed by

(6) $$c_{e}=\frac{K^{2}}{R_{coil}+R_{load}}$$

Normally, when looking for the maximum power transfer, the theory of impedance matching is applied. By using this technique, the optimum load resistance is claimed to be equal to the coil resistance. However, Stephen et al. [34] demonstrated that this approach is incorrect for the electromagnetic harvester system, since it does not take into account the effect of electrical damping on the mechanical behavior. He proposed a more precise method to obtain the optimal load resistance value, which includes the electrical analogue of the mechanical damping. Thus, the optimum load resistance that maximizes the output power is defined as

(7) $$R_{load,opt}=R_{coil}+\frac{K^{2}}{c_{m}}$$

Equations (1) and (2) can be solved numerically to obtain the value of the load voltage *V* and the load power *P* of the system. The voltage across the load resistance and the corresponding output power are given by

(8) $$V=K\dot{z}\frac{R_{load}}{R_{load}+R_{coil}}$$

and

(9) $$P=\frac{V^{2}}{R_{load}}$$

### 2.2. Coil–Magnet Structure

**Figure 2:**
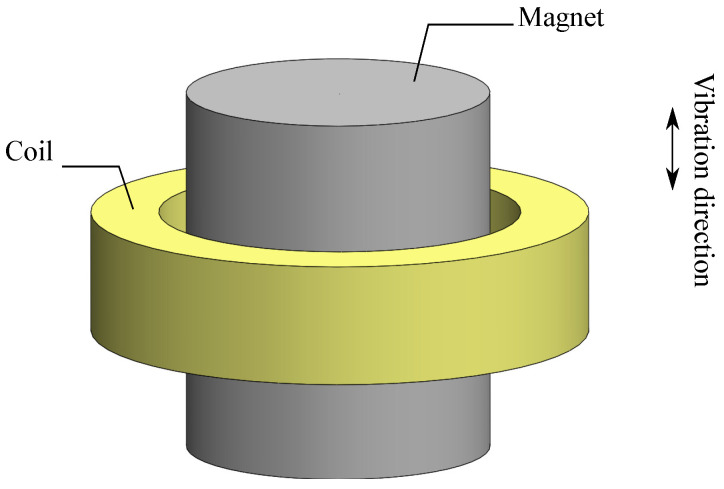
Basic arrangement of a magnet in-line coil structure with the cylindrical magnet and hollowed, cylindrical coil.

The electromagnetic energy harvester can be implemented using different electromagnetic coupling architectures. These are classified based on the arrangement of the magnet and coil. There are two main coil–magnet architectures, including magnet in-line coil architecture and magnet across coil architecture [26]. The first one refers to the structures where the center axis of magnet and coil is in line with the oscillation direction. The second one, on the other hand, implies structures where the center axis of magnet and coil is orthogonal to the oscillation direction. This study is limited to the structures that belong to the first class. Specifically, the structures with cylindrical magnets inside cylindrical shaped coils are under investigation. The basic arrangements of these types of structures are shown in Figure 2. The number of the coils and magnets will be different for individual cases. Since the spring and housing can be implemented in many different ways, the construction volume in the study only considers the coil–magnet structure volume. The coils investigated in this study are wire-wound coils with multi-layer and circular shape. Assuming a wire-wound coil with a given inner radius $r_{i}$, outer radius $r_{o}$, and thickness *t*, we can express the coil parameters, such as coil resistance $R_{c}$ and number of turns *N* such that

(10) $$R_{c}=\frac{\rho_{c}N^{2}\pi \left(r_{o}+r_{i}\right)}{k_{c}\left(r_{o}-r_{i}\right)t},$$

(11) $$N=\frac{4k_{c}\left(r_{o}-r_{i}\right)t}{\pi d^{2}},$$

where $\rho_{c}$ refers to the coil resistivity, $k_{c}$ is the filling factor of the coil and *d* is the diameter of the coil.

In the magnetic field of a coil–magnet structure, the total magnetic flux passing through the loops of the coil is called flux linkage. Generally, the total flux linkage for a coil with multiple turns is calculated based on the linkage for the individual coil turns with

(12) $$\phi =\sum_{i=1}^{N}\int_{A_{i}}^{}B\;\cdot \;dA,$$

where *N* is the number of coil turns, *B* is the magnetic flux density over the area of the *i*th turn. Usually, the flux density can be considered uniform over the coil area. In such cases, the total flux linkage can be rewritten as

(13) ϕ=NBAsin(α),

where α is the angle between the flux density direction and the coil area. When the magnets and coil move relative to each other, the flux linkage is changed and thus an output voltage is induced. Therefore, for the electromagnetic energy harvester, the flux gradient, which is the time rate of change of the magnetic flux linkage, is a more relevant term. This value, often referred to as the electromagnetic coupling coefficient *K*, is the link between the mechanical and electrical domains, and is expressed as

(14) $$K=\frac{d\phi }{dz}$$

## 3. Design Optimization

In this section, the parameters of the magnetic structure are designed and optimized to yield the maximum output power. A single process to obtain the output power from a certain coil–magnet geometry consists of two steps. First, a finite element method (FEM) simulation software is used to extract the electromagnetic coupling factor from the magnetic structure. Then, using the obtained coupling factor, the output power is calculated using the differential equations given in (1) and (2). In this work, ANSYS Maxwell and MATLAB Simulink are used for these two stages, respectively. Each pair of design parameters and corresponding output power is considered as input and output of an expensive black-box function f(x), which refers to a time consuming simulation model whose analytical description is not available. Thus, the problem of optimizing for output power can now turn into an expensive black-box optimization. The optimization method, design parameters, and implementation procedure are presented in details as follows.

### 3.1. Optimization Method

**Figure 3:**
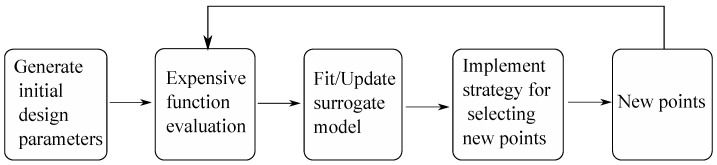
Flow chart of an expensive black-box optimization algorithm with surrogate model. Adopted and generalized from [38].

A popular approach for solving optimization problems is to first approximate the black-box function using response surface models and then to utilize these models to search for the optimal solutions. These methods are often referred to as surrogate-based optimization methods. Response surface models, which are also known as surrogate models or metamodels, are simply inexpensive approximate models to determine promising points for function evaluation. Some of the models used in this optimization method include, but are not limit to, polynomials [35], radial basis functions (RBF) [36], Kriging models [37] and neural networks. While polynomials are limited to linear and quadratic cases, RBF and Kriging can fit higher-degree functions. Furthermore, these two models are relatively simple to build compared to neural networks. Thus, they are popular models used in the black-box function optimization approach. In this study, a RBF-based method for global optimization of expensive functions proposed by Regis et.al [38] is adopted and implemented. The steps of this optimization method have been generalized into the flow chart shown in Figure 3.

Latin hypercube sampling (LHS) [39] is used to generate initial random sample parameters, which are limited by lower and upper boundaries. This step aims to spread the sample points evenly across all possible values of box-constrained design parameters. The range of each design parameter is first divided into n sections, where n is the number of sampling points. In the current algorithm, this number is defined to be 2(d + 1) where d is the number of design parameters. As a next step, one point is placed in a randomized location within its section. Further, the construction of the LHS design can be controlled using different criteria, such as centering the points within the sampling intervals, maximizing the minimum distance between points, and minimizing the minimum correlation coefficient. In our algorithm, the criterion of maximizing the minimum distance between points (maximin) is implemented. Specifically, a number of LHS samples is generated at random iteratively and the one that satisfies the criterion ‘maximin’ will be chosen. Next, the simulation is called to evaluate the expensive function at the generated points. The point with the best function value obtained from the simulation is set as the best point and the surrogate model is fitted or updated based on the simulated values of its function evaluation. During these steps, the radial basis function (RBF) interpolation model is used as the surrogate model and ANSYS Maxwell and MATLAB Simulink are used for simulation. In the next step, a set of candidate points for the next evaluation is generated by applying normally distributed perturbations on some of the coordinates of the current best point. Then, the next evaluated point is selected based on the information from the response surface model and all the previous evaluated points. Specifically, the candidate point that results in the best estimated function value from the RBF surrogate, and that is also far from previous evaluated points, should be chosen for the next iteration. For each iteration, the process of function evaluation and surrogate model update are repeated again for new selected points and new points are being generated until the stop criterion is met. The stop criterion that is adopted in the current algorithm, is a maximum number of allowed function evaluations.

### 3.2. Design Parameters

**Figure 4:**
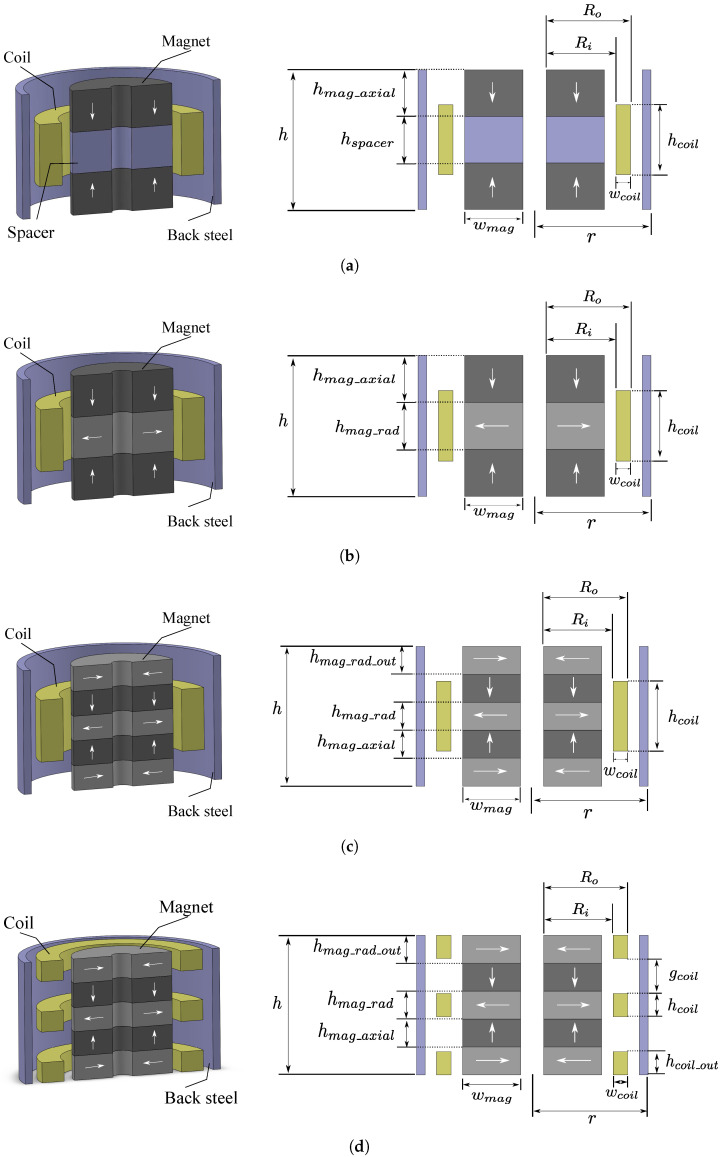
Magnetic structure of the investigated cylindrical electromagnetic energy harvesters: (**a**) Configuration DM1S1C. (**b**) Configuration H3M1C. (**c**) Configuration H5M1C. (**d**) Configuration H5M3C. Right-hand side depicts a 2D representation with relevant dimensions.

In this study, four coil–magnet configurations are investigated and compared in terms of output power performance of the corresponding harvesters. The details of each configuration are shown in Figure 4. As shown in the figure, these configurations have different number of magnets and coils, as well as different magnet arrangements. The first configuration (DM1S1C) has two magnets with like-poles facing each other and a mild steel used as a spacer, which aims to concentrate the magnetic field flow. Only one coil is used in this configuration. The other configurations utilize Halbach arrays in the magnetic structure with different numbers of magnet. These configurations are a Halbach array with three magnets and one coil (H3M1C), a Halbach array with five magnet and one coil (H5M1C), and a Halbach array with five magnets and three coils (H5M3C). All the coil–magnet structures are surrounded by a back shield, which guides the magnetic flux and minimizes the flux leakage. The magnetic field line direction inside the magnet is indicated as an arrow with the direction from south to north. We refer to the magnets as radial magnets or axial magnets depending on whether their direction is in line with the harvester’s axis or orthogonal to it. Since the configurations are symmetrical, the coil (in case of one-coil configuration) or the center coil (in case of multi-coil configuration) is aligned with either the spacer or the center radial magnet for the best efficiency.

A number of fixed harvester parameters and dimensions in this study are adopted from Ordoñez et al. [33]. This allows for a direct comparison of the results among the two studies. Consequently, all configurations are constrained in a volume of 30.15 $cm^{3}$, which has a cylindrical shape with fixed base radius r=20 mm and fixed height h=24 mm. Other parameters that are kept constant are coil properties, back shield parameters, magnet inner radius, the gap between the coil and magnets, and the gap between the magnet and back steel. These parameters and their values are listed in Table 1. The magnet material is NdFeB-N52 and the steel 1010 is used as material for spacer and back shield. The harvester configurations are then subjected to the same excitation conditions described in Table 2 and the output power is maximized for each harvester to result in a fair comparison.

The design parameters of the harvester will be used as the input for the optimization process. Thus, for each configuration of coils and magnets, a set of design variables is defined based on the parameters that we want to optimize. As can be seen from Figure 4, the distance between magnet inner radius and coil inner radius is denoted $R_{i}$, and the distance between magnet inner radius and coil outer radius is denoted $R_{o}$. Other geometrical dimensions are named correspondingly in the figure. As a result, the possible design parameters include the ratio of $R_{i}$ to $R_{o}$ ($R_{i}/R_{o}$), the ratio of the height of the spacer to the height of the total volume ($h_{spacer}/h$), the ratio of the height of the coil to the height of the total volume ($h_{coil}/h$), the ratio of the height of radial magnets to the height of the total volume ($h_{mag\_rad}/h$), the ratio of the total height of all radial magnets to the height of the total volume ($h_{mag\_rad\_total}/h$), the ratio of the height of the outer radial magnet to the height of the center radial magnet ($h_{mag\_rad\_out}/h_{mag\_rad}$), the ratio of the total height of the three coils to the height of the total volume ($h_{coil\_total}/h$), the ratio of the height of the outer coil to the height of the center coil ($h_{coil\_out}/h_{coil}$), and the ratio of the distance between the outer coil and the center coil to the maximum distance between them ($g_{coil}/g_{coil\_max}$). This maximum distance is defined as the distance between the outer coil and the center coil when the outer coil is placed at the outer boundary of the total volume. Based on geometrical the structure and its limitations, different configurations utilize different sets of these design variables for the optimization process, and these parameters are constrained with upper and lower bounds, as shown in Table 3.

### 3.3. Implementation Procedure

**Figure 5:**
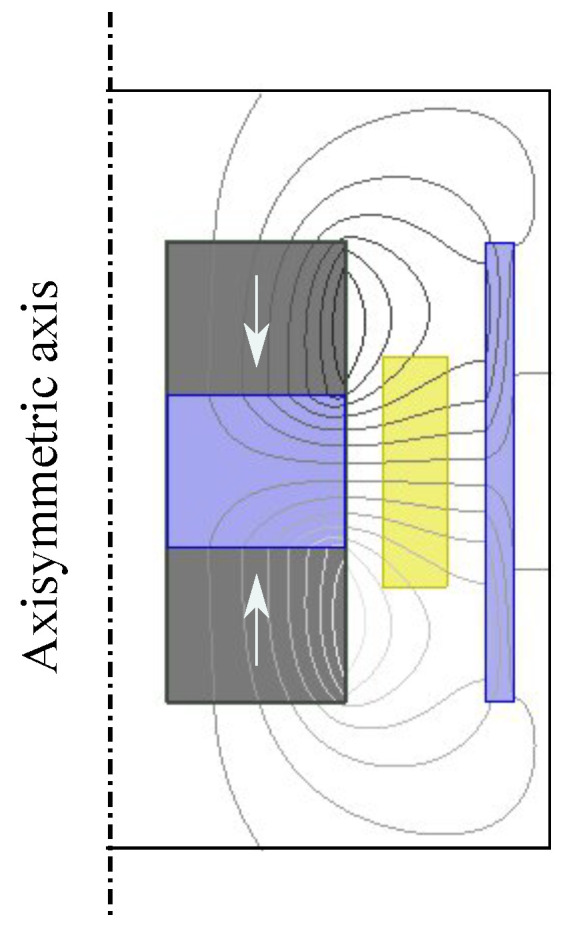
Exemplified model implementation in ANSYS Maxwell. The models are implemented in 2D and utilize the axisymmetric property of the configurations to result in time efficient simulations.

As presented at the beginning of this section, the first step in the process of obtaining output power from the harvester is to deal with the magnetic properties of the coil–magnet structure. In this work, the software tool ANSYS Maxwell is used to extract the electromagnetic coupling coefficient from each design. In order to reduce simulation time and improve meshing precision, a two dimensional (2D) axisymmetric model of each coil–magnet configuration is built in the software as shown in Figure 5. The total magnetic flux linkage is then generated by making the parts move relative to each other using motion setup in the model. When all the settings are completed, the simulation is carried out to obtain a plot of the magnetic flux linkage against the displacement between coils and magnets. The coupling coefficient is then extracted from the curve with a polynomial fitting function.

**Figure 6:**
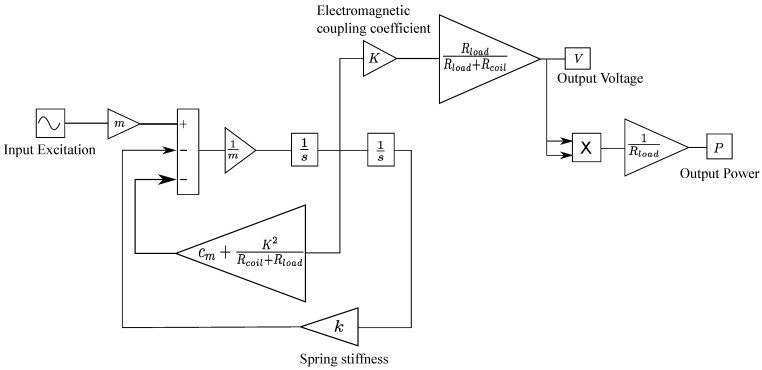
Implementation of the differential equations of the electromagnetic energy harvester for output power calculation.

In order to obtain the output power, a Simulink model is set up in MATLAB implementing the differential equations provided in Section 2. The model implementation can be seen in Figure 6. In the model, there are two types of input parameters. A set of parameters is constant for all simulations, whereas others are changing according to the change of design parameters. Specifically, the vibration conditions are kept constant (see Table 2) while the values of mass, spring stiffness, electromagnetic coupling coefficient, load resistance, and coil resistance need to be adjusted to adapt with the changing design parameters.

The two modeling steps outlined above are applicable for a single process to acquire the output performance from a certain set of design parameters. In our optimization process, these steps are repeated multiple times and the selection of input design values for each iteration depends on the output value obtained from the preceding iteration. Thus, an overarching environment that can interface with both ANSYS Maxwell and MATLAB Simulink is needed to manage the optimization algorithm. This functionality is provided by the Python implementation proposed in [40] for the optimization algorithm described in Section 3.1. Additionally, the Pythonic interface PyAEDT [41] is employed to enable the usage of ANSYS products through Python.

**Figure 7:**
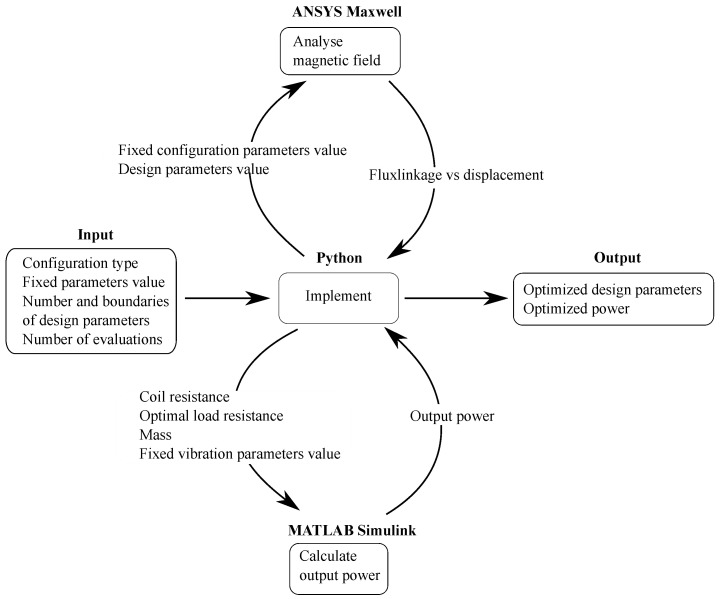
Interaction diagram between the different tools of the multi-tool optimization process. ANSYS Maxwell and MATLAB Simulink are called directly from the Python environment, and thus the user only interacts with Python.

The implementation of the whole procedure is outlined in Figure 7. The optimization process can be executed in a fully automated manner, with no requirements on users to adjust the design parameters manually. Users input a set of desirable parameters in order to configure the optimization process. These parameters include (i) the configuration type to guide the optimization system to select the right model for the investigation; (ii) fixed configuration and vibration parameters to input to Maxwell and Simulink models, respectively; (iii) the number of design parameters and corresponding boundaries to facilitate the steps of generating initial design parameters and creating new points; and (iv) the number of evaluations as a stop criterion. Afterwards, users only need wait to obtain the optimized output values. In the Python environment, the design parameter values are first initiated and then sent to Maxwell for magnetic field analysis. Then the result plot of flux linkage versus displacement is routed back to Python for electromagnetic coupling coefficient calculation. The computed coupling coefficient, together with other variables needed for the Simulink model, are then sent to Simulink for power calculation and the result is fed back to Python. The optimization implementation in Python will then continue based on the obtained output power from Simulink and current design parameter values. The procedure is repeated until the specified number of iterations is reached.

## 4. Results and Discussions

### 4.1. Validation

**Figure 8:**
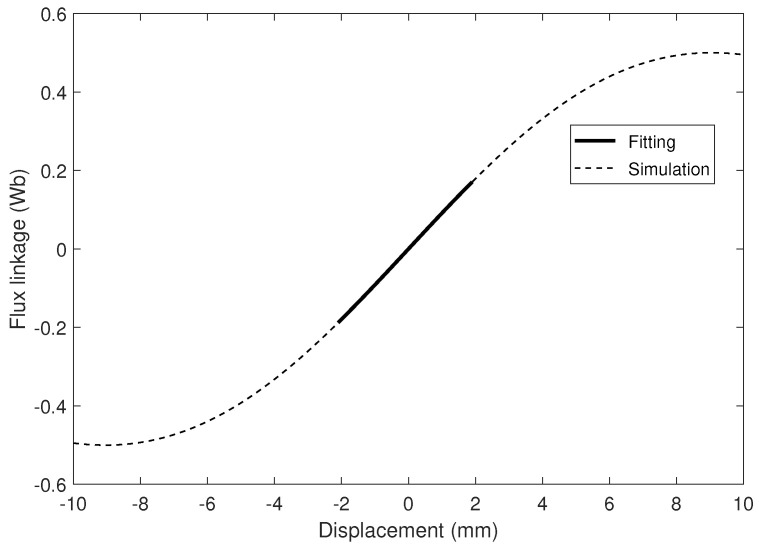
The relationship between the flux linkage and the displacement for the H3M1C configuration with parameters listed in Table 4.

In order to validate the proposed process of obtaining the output power, an initial investigation was conducted with a configuration and parameters adopted from Ordoñez et al. [33]. For the validation, the configuration with a three-magnet Halbach array and one coil was chosen. The parameters for this configuration are listed in Table 4. By using ANSYS Maxwell, we obtain the corresponding flux linkage - displacement curve as exemplified in Figure 8. The simulated flux linkage over a displacement range from −10 mm to 10 mm was shown in the dashed black line. Since we only consider small oscillation amplitudes, a fitting function was applied to the simulation curve over a smaller range of displacements. The electromagnetic coupling coefficient was then extracted from the fitted curve, which is shown as a solid line in the figure. Finally, the output power can be calculated with the Simulink model.

Table 5 provides a comparison between the simulation results from our study and the results of Ordoñez et al. [33]. As can be seen from the table, these simulation results are very similar to each other, verifying the validity of our implementation. The small variation between the results are likely a consequence of the difference in simulation software used for magnetic field analysis.

### 4.2. Power Optimization

**Figure 9:**
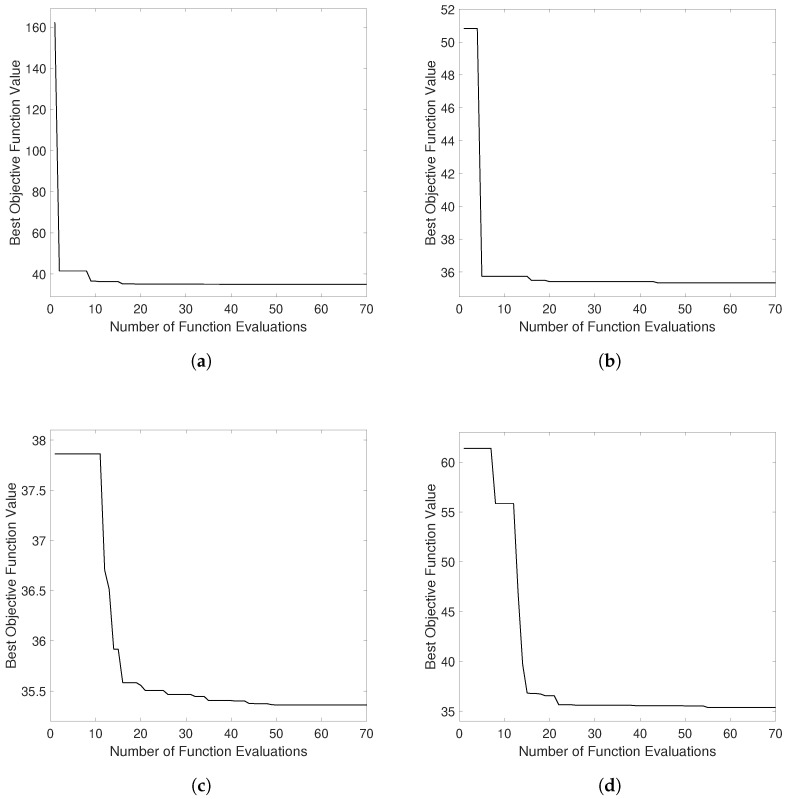
Best objective function value vs. number of function evaluation for different configurations: (**a**) Configuration DM1S1C. (**b**) Configuration H3M1C. (**c**) Configuration H5M1C. (**d**) Configuration H5M3C. The objective function is the inverse of the output power.

The next investigation is to optimize the output power of the four harvester configurations based on variable design parameters. Figure 9 shows the plots of the best objective function value versus the number of iterations for each configuration. The objective function value in our study was set to be the inverse of the output power. Thus, the optimization target is to minimize the objective function. The maximum number of iterations was set to 70, and it can be seen that the optimization algorithm converges after a certain number of function evaluations. The converged values are 34.939, 35.337, 35.360, and 35.365, respectively.

**Figure 10:**
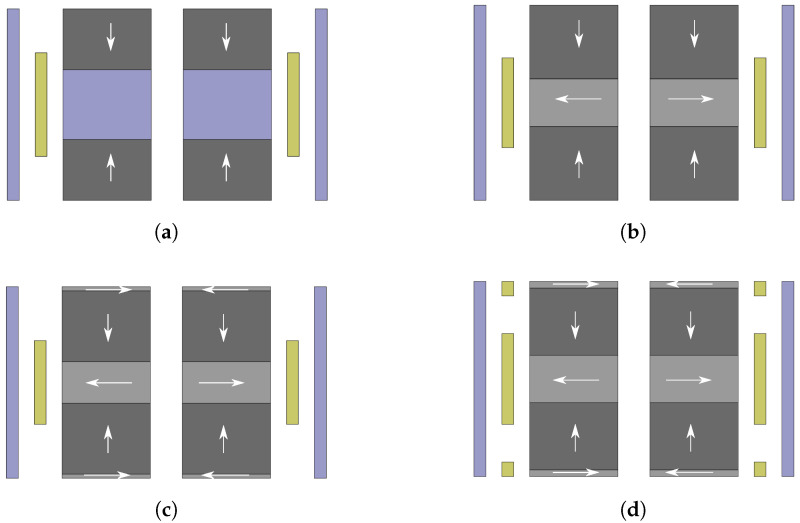
Resulting structures of the four configurations after output power optimization: (**a**) Configuration DM1S1C. (**b**) Configuration H31C. (**c**) Configuration H5M1C. (**d**) Configuration H5M3C.

Table 6 presents the optimal design values for each configuration, while Figure 10 shows the visualization of the resulting optimal geometries. From these, we can see that the geometries of the third and the fourth configuration tend to converge to the geometry of the second configuration. In other words, the optimization algorithm adjusts the configurations of H5M1C and H5M3C to become as close as possible to H3M1C in order to optimize for power.

Table 7 lists the maximum output power for each design and the corresponding value of the output voltage, the volume power density and the mass power density. These results show that the Halbach array with three magnets and one coil can produce an optimal output power of 28.3 mW, which is approximately 18% higher than the output of the non-optimized case investigated in [33]. Thus, the optimization procedure proves to be effective with respect to the performance of the harvester. Moreover, the optimal output power values of the three Halbach array configuration are quite similar, which is a result of the fact that all of these configurations seem to converge to become the same configuration (H3M1C). It can also be noticed that the maximum output power of the H3M1C configuration is slightly higher than that of the H5M1C configuration. This is due to the space requirements of the additional outer radial magnets for H5M1C configuration, which reduces the height of axial magnets and the middle radial magnet of this configuration. Finally, the dual-magnet array with spacer demonstrates a higher performance as the other configurations in terms of the maximum output power and the volume power density, while the Halbach array with three magnet and one coil appears to be the best option when the mass power density is considered. Only small difference in terms of performance can be observed between these two configurations. The reason may be due to the magnetic flux in these two cases having similar direction, and due to the mass density of steel being somewhat higher than that of the permanent magnet.

### 4.3. Electromagnetic Coupling Coefficient Maximization

**Figure 11:**
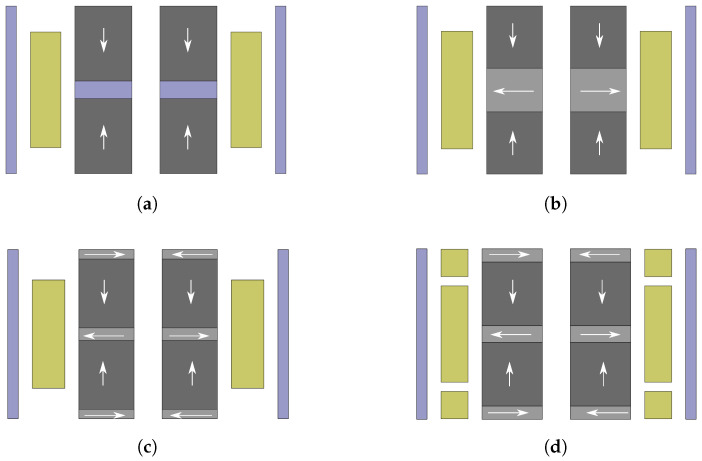
Resulting structures of the four configurations after electromagnetic coupling maximization: (**a**) Configuration DM1S1C. (**b**) Configuration H3M1C. (**c**) Configuration H5M1C. (**d**) Configuration H5M3C.

In many previous works, the focus was on the electromagnetic coupling coefficient, with the aim to enhance output power [42,43]. This approach would avoid output power estimation in each iteration and, thus, motivates an investigation of the relation between these two optimization goals. In order to perform this investigation, the objective function was replaced by the inverse of the coupling coefficient and the same optimization procedure as outlined before was applied. Table 8 lists the optimized design parameters of the four configurations with respect to a maximized coupling coefficient. Figure 11 depicts the corresponding structures. The obtained coupling coefficients, as well as the corresponding output power and output voltage values, are also included in the table.

It is shown that the Halbach array, with five magnets and three coils (H5M3C), has the highest coupling coefficient of the investigated configurations. However, it is also shown that maximizing the coupling coefficient does not mean maximize the output power. The output power values obtained when maximizing the coupling coefficient are significantly lower than the previously achieved output power values. The likely reason for this is that the output power optimization process in this study takes into account all the design parameters related to the positions and dimensions of coils and magnets, such as coil width, coil height, distance between coils, magnet width, and magnet height. The change in these parameters will lead to a change in the coil resistance and the moving mass, which affect the optimal load resistance and the relative velocity between the magnets and the coil. These factors need to be considered together with the value of the coupling coefficient to determine the optimal output power.

## 5. Conclusions

In this paper, we investigated and compared the performance of four cylindrical coil–magnet configurations for electromagnetic vibration energy harvesters. These prototypes are based on either cylindrical Halbach arrays or opposite polarized magnets. An expensive black-box optimization algorithm was implemented to maximize the output power of these harvester configurations under a fixed volume constraint. All of the parameters regarding the dimensions and locations of the magnets and coils were under the scope of investigation. ANSYS Maxwell and MATLAB Simulink were utilized to facilitate the analysis process.

Implementing an automated optimization approach remarkably simplified the optimization procedure. Requirements for manual interference were completely eliminated. By using this approach, users no longer need to select and evaluate random sampling manually, or use intuition to find the best solution. In comparison to previous studies that investigated non-optimized structures, we demonstrated that optimization achieves a significant performance improvement.

The simulation results show that the Halbach array configuration with five magnets and three coils (H5M3C) and the Halbach array configuration with five magnets and one coil (H5M1C) tend to converge to the Halbach array configuration with three magnets and one coil (H3M1C) during the design optimization procedure. This indicates that H3M1C seems to be a better candidate for Halbach array configurations under the constraints and excitation conditions investigated in this study.

Among all configurations, the configuration with two opposite polarized magnets (DM1S1C) presents a slightly higher performance in terms of output power and volume power density than the others. However, the Halbach array with three magnets and one coil (H3M1C) provides the highest mass power density.

It is also observed that for output power optimization, where all the design parameters were under consideration, not only the coupling coefficient, but also other aspects that vary with the change of the design parameters, will affect the final performance result. Thus, the electromagnetic coupling coefficient should not be used as a metric to optimize an energy harvester if the ultimate goal is to maximize output power.

The automated optimization method, results, and conclusions of this study are significant contributions to the optimization and comparison of electromagnetic vibration energy harvesters. As such, they have the potential to result in more effective harvester implementations that lead to improved utility in a number of application scenarios.

The current study focuses on the optimization and comparison of electromagnetic energy harvesters with different cylindrical magnet–coil structures under a specific volume constraint and subjected to a specific set of vibration conditions. In addition, since the black-box optimization approach takes into account multiple parameters that can affect the output results, this method is suitable for problems that require complicated computation or are difficult to be modeled in an analytical manner. Thus, this approach can give an initial idea on the output of the complicated system in terms of a practical implementation. The lacking ability of this approach to link the result to the underlying theory, however, can be seen as a limitation of the proposed approach. With the goal of generalization, further investigations should be performed to study the optimization procedure in additional scenarios, with variations in volumetric constraints and excitation conditions. Moreover, future research on a theoretical model of the current approach could be an interesting topic.

## Figures and Tables

**Table 1:** Constant parameters of the electromagnetic energy harvester based on dimensions and materials according to [33].

Parameters	Values
Constrained volume	30.15 cm${}^{3}$
Constrained height	24 mm
Constrained base radius	20 mm
Gap between coil and magnet	2 mm
Gap between coil and back shield	2 mm
Magnet inner radius	2 mm
Back shield thickness	1.5 mm
Back shield height	24 mm
Density of magnet	7500 kg m${}^{-3}$
Density of steel 1010	7872 kg m${}^{-3}$
Coil wire diameter	0.1 mm
Coil fill factor	0.65

**Table 2:** Vibration conditions used as excitation of the energy harvesters.

Parameters	Values
Excitation amplitude (RMS)	0.2 g
Frequency of vibration	55 Hz
Mechanical damping ratio	0.00457

**Table 3:** Relevant design parameters of the harvester configurations, as well as their upper and lower bounds.

Design Parameter	Configurations	Lower Boundary	Upper Boundary
*R_i_/R_o_*	DM1S1C, H3M1C, H5M1C, H5M3C	0.2	0.9
*h_spacer_/h*	DM1S1C	0.1	0.9
*h_coil_/h*	DM1S1C, H3M1C, H5M1C		
*h_mag_rad_/h*	H3M1C		
*h_mag_rad_total_/h*	H5M1C, H5M3C		
*h_coil_total_/h*	H5M3C		
*h_mag_rad_out_/h_mag_rad_*	H5M1C, H5M3C	0.1	1
*h_coil_out_/h_coil_*	H5M3C		
*g_coil_/g_coil_max_*	H5M3C		

**Table 4:** Parameters of Halbach array configuration with three magnets and one coil (H3M1C) for verification. Parameters are selected in accordance with [33] to evaluate model implementation.

Parameters	
Magnet outer radius	12 mm
Magnet height	8 mm
Moving mass	79.2 g
Coil inner radius	14 mm
Coil outer radius	16.5 mm
Coil height	12 mm
Coil number of turns	2483
Coil resistance	510 Ω
Remnant flux density	1.45 T
Coercive field strength	976 kA/m

**Table 5:** Verification results of the non-optimized H3M1C configuration with previously reported results according to [33].

	Our Work	Ordoñez et al. [33]
Optimal load resistance (kΩ)	34.3	37.55
Coupling coefficient (Wb m^−1^)	91.94	95.83
Output power (mW)	23.95	23.8

**Table 6:** Design parameters of the four harvester configurations after output power optimization.

	Configuration	Configuration	Configuration	Configuration
	DM1S1C	H3M1C	H5M1C	H5M3C
*R_i_/R_o_*	0.899	0.899	0.899	0.899
*h_coil_/h*	0.541	0.46	0.437	-
*h_coil_total_/h*	-	-	-	0.614
*h_coil_out_/h_coil_*	-	-	-	0.16
*h_spacer_/h*	0.364	-	-	-
*h_mag_rad_/h*	-	0.245	-	-
*h_mag_rad_total_/h*	-	-	0.261	0.311
*h_mag_rad_out_/h_mag_rad_*	-	-	0.1	0.14
*g_coil_/g_coil_max_*	-	-	-	0.99
*w_mag_* (mm)	11.0355	11.0355	11.0355	11.0355
*h_spacer_* (mm)	8.736	-	-	-
*h_mag_rad_* (mm)	-	5.88	5.22	5.83
*h_mag_rad_out_* (mm)	-	-	0.522	0.816
*h_mag_axial_* (mm)	7.632	9.06	8.868	8.269
*w_coil_* (mm)	1.46	1.46	1.46	1.46
*h_coil_* (mm)	12.984	11.04	10.488	11.16
*h_coil_out_* (mm)	-	-	-	1.78
*g_coil_* (mm)	-	-	-	4.58
*N_coil_turn_*	1573	1338	1271	1353
*R_coil_* (Ω)	333	284	269	296
*N_coil_out_turn_*	-	-	-	216
*R_coil_out_* (Ω)	-	-	-	41
*R_load_opt_* (kΩ)	13.2	14.9	13.7	17.6

**Table 7:** Performance comparison of the different configurations after output power optimization.

	Configuration	Configuration	Configuration	Configuration
	DM1S1C	H3M1C	H5M1C	H5M3C
Output power (mW)	28.62	28.3	28.28	28.278
Output voltage (V)	19.4	20.55	19.7	22.26
Volume power density (mW cm^−3^)	0.949	0.939	0.938	0.938
Mass power density (mW g^−1^)	0.299	0.3018	0.3015	0.3015

**Table 8:** Design and performance parameters of the four configurations after optimization for maximum electromagnetic coupling.

	Configuration	Configuration	Configuration	Configuration
	DM1S1C	H3M1C	H5M1C	H5M3C
*R_i_/R_o_*	0.7	0.69	0.68	0.734
*h_coil_/h*	0.69	0.7	0.64	-
*h_coil_total_/h*	-	-	-	0.887
*h_coil_out_/h_coil_*	-	-	-	0.285
*h_spacer_/h*	0.106	-	-	-
*h_mag_rad_/h*	-	0.26	-	-
*h_mag_rad_total_/h*	-	-	0.187	0.254
*h_mag_rad_out_/h_mag_rad_*	-	-	0.751	0.79
*g_coil_/g_coil_max_*	-	-	-	0.956
*w_mag_* (mm)	8.15	8	7.86	8.64
*h_spacer_* (mm)	2.54	-	-	-
*h_mag_rad_* (mm)	-	6.24	1.79	2.36
*h_mag_rad_out_* (mm)	-	-	1.35	1.86
*h_mag_axial_* (mm)	10.73	8.88	9.75	8.96
*w_coil_* (mm)	4.35	4.5	4.64	3.86
*h_coil_* (mm)	16.56	16.8	15.36	13.56
*h_coil_out_* (mm)	-	-	-	3.86
*g_coil_* (mm)	-	-	-	1.3
*N_coil_turn_*	5962	6250	5898	4328
*R_coil_* (Ω)	1148	1197	1124	847.6
*N_coil_out_turn_*	-	-	-	1233
*R_coil_out_* (Ω)	-	-	-	241.6
*R_load_opt_* (kΩ)	74.2	81.5	63.6	81.68
Coupling coefficient (Wb m^−1^)	115.07	117.65	100.15	125
Output voltage (V)	35.5	36.6	31.78	39
Output power (mW)	16.82	16.74	15.89	18.65

## Data Availability

Not applicable.
